# Walking against or with traffic? Evaluating pedestrian fatalities and head injuries in Taiwan

**DOI:** 10.1186/s12889-019-7588-1

**Published:** 2019-10-10

**Authors:** Chih-Wei Pai, Ping-Ling Chen, Shiao-Tzu Ma, Shan-Hong Wu, Václav Linkov, Hon-Ping Ma

**Affiliations:** 1 Graduate Institute of Injury Prevention and Control, College of Public Health, Taipei Medical University, Taipei, Taiwan; 2 Department of Emergency Medicine, Taipei Municipal Wanfang Hospital, Taipei Medical University, Taipei, Taiwan; 3 Department of Traditional Chinese Medicine, Taipei Medical University Hospital, Taipei, Taiwan; 4 Department of Traffic Psychology, CDV - Transport Research Centre, Brno, Czech Republic; 5 Department of Emergency Medicine, Shuang-Ho Hospital, Taipei Medical University, Taipei, Taiwan; 6 Department of Emergency Medicine, School of Medicine, Taipei Medical University, Taipei, Taiwan

**Keywords:** Pedestrian crash, Walking against traffic, Walking with traffic, Fatalities, Head injuries

## Abstract

**Background:**

Allowing contraflow cycling on one-way streets has been reported to reduce crash risks in Belgium and the United Kingdom. Similarly, walking against traffic on roadways without sidewalks substantially improves pedestrian safety. This study examined fatalities and head injuries sustained by pedestrians in against-traffic and with-traffic crashes.

**Methods:**

Using police-reported crash data in Taiwan between 2011 and 2016, fatalities and head injuries were compared for pedestrians involved in against-traffic and with-traffic crashes.

**Results:**

Of the 14,382 pedestrians involved in crashes, 10,749 and 3633 pedestrians in with-traffic and against-traffic crashes, respectively, were reported. Compared with pedestrians involved in against-traffic crashes, those in with-traffic crashes were more likely to sustain fatalities and head injuries. Results of logistic regression models revealed several influential factors on pedestrian fatalities and head injuries, including elderly pedestrians, male drivers, intoxicated drivers, rural roadways, unlit streets in darkness, limited sight distance, adverse weather conditions, midnight hours, and a heavy vehicle as the crash partner.

**Conclusions:**

Pedestrians in with-traffic crashes were more likely to sustain fatalities and head injuries compared with those in against-traffic crashes. Furthermore, the negative effect of walking with traffic on injuries was more pronounced in reduced-visibility conditions.

## Background

Contraflow cycling permits cyclists to travel in the opposite direction of oncoming motorised vehicles on one-way street [1]. Contraflow cycling schemes on one-way streets have been reported to reduce crash risks in European countries, notably in the United Kingdom [2] and Belgium [3]. The primary reason for such a beneficial effect of contraflow cycling schemes is that motorists and cyclists can maintain continuous eye contact and execute evasive manoeuvres to avoid crashes. In addition, motorists may be highly alert to oncoming cyclists because they generally consider contraflow cycling to be dangerous [4]. For roadways without contraflow traffic management scheme, cycling in the wrong direction was the leading cause of head-on crashes [5].

Although not mandatory in general, several States such as Florida [6] has enacted law mandating pedestrians to walk against oncoming traffic on roadways without sidewalks. The primary reason for this law is that, similar to contraflow cycling schemes, motorists and pedestrians can perceive each other. Research [7] has reported that pedestrians walking against traffic appeared to be more conspicuous, particularly at nights, compared with those walking with traffic; they speculated that walking against traffic may provide a biological motion advantage that reminds drivers of the presence of pedestrians. Evidence from other studies may support law mandating pedestrians to walk against traffic. For example, a study [8] conducted in France reported that of 100 fatally-injured pedestrians walking along on the streets, 75 were walking with traffic. A recent study [9] conducted in Finland examined pedestrian crashes and suggested that, compared with walking with traffic, walking against traffic resulted in a 77% decrease in the number of pedestrian crashes. Such a beneficial effect of walking against traffic is attributable to the visual information it provides regarding vehicles in the lane closest to pedestrians. Han et al. [10] and Liu et al. [11] have reported that pedestrians who were struck from behind were more likely to sustain head injuries, which are normally devastating or fatal.

Other crash types involving pedestrians at mid-blocks include nearside (right side, the side of vehicle nearest the kerb) and offside (left side, the side of vehicle nearest the central line) crashes. Elderly pedestrians were found to be over-involved in offside crashes [12]. This is primarily because they walk more slowly than their younger counterparts, and thus have difficulties crossing roadways before traffic signals change [12]. Furthermore, as older pedestrians have diminished attention capacity, they are less capable of judging two-stream traffic before crossing roadways compared to younger pedestrians. Children, on the other hand, tend to exhibit poor navigational capability and enter roadways without considering traffic sensibly; as a result, children were found to be over-involved in nearside crashes [13].

In Taiwan, pedestrians are advised to walk facing traffic, mainly because this has been suggested to increase pedestrian safety. Official crash statistics in year 2017 revealed that crashes involving pedestrians walking along the street account for approximately 10% of all mid-block crashes that involved pedestrians and other motorised vehicles [14]. Injuries sustained by pedestrians in this crash type appear to be a safety concern because crash velocities tend to be higher than those occurring at intersections [15].

When reviewed together, literature has suggested that contraflow cycling schemes or walking against traffic may reduce crash risks. To our knowledge, relatively few studies have investigated whether the beneficial effect of walking against traffic on crash risks can also apply to injury severity.

### Purpose

Following the pioneering study conducted in Finland [9] that has concluded that walking against traffic is beneficial in reducing pedestrian crashes, the primary aim of this study was to examine injury severity and head injuries sustained by pedestrians in facing-traffic and back-to-traffic crashes.

## Methods

### Data source

Using the Taiwan National Traffic Crash Dataset for the period 2011–2016, the current study examined fatalities and head injuries sustained by pedestrians in against- or with-traffic crashes. The Taiwan National Traffic Crash Dataset is owned and maintained by the National Police Agency, and the data are recorded after every road traffic crash is reported to the police. To record crash data, qualified and experienced police crash investigators complete three files, namely accident, vehicle, and victim files. Accident files contain general information regarding an accident, such as the time and date of the crash, weather condition, and road type. Vehicle files contain information regarding vehicle type, the first point of impact, and vehicle manoeuvres. Victim files contain data regarding casualties involved in crashes, such as age, sex, injury severity, injured body regions, licence status, walking direction, and alcohol use. Injury severity is classified into two categories: fatality and injury. In the victim file, pedestrians walking directions, namely crossing a street, walking against or with traffic, are recorded. Victims who die within 24 h as a result of an accident are classified as cases of fatality, whereas victims who sustain injuries, whether mild or severe, are classified as cases of injury. Crash investigators track injury data from hospitals for 30 days and thus obtain data on primary diagnosis on injured body regions and injury severity.

**Fig. 1:**
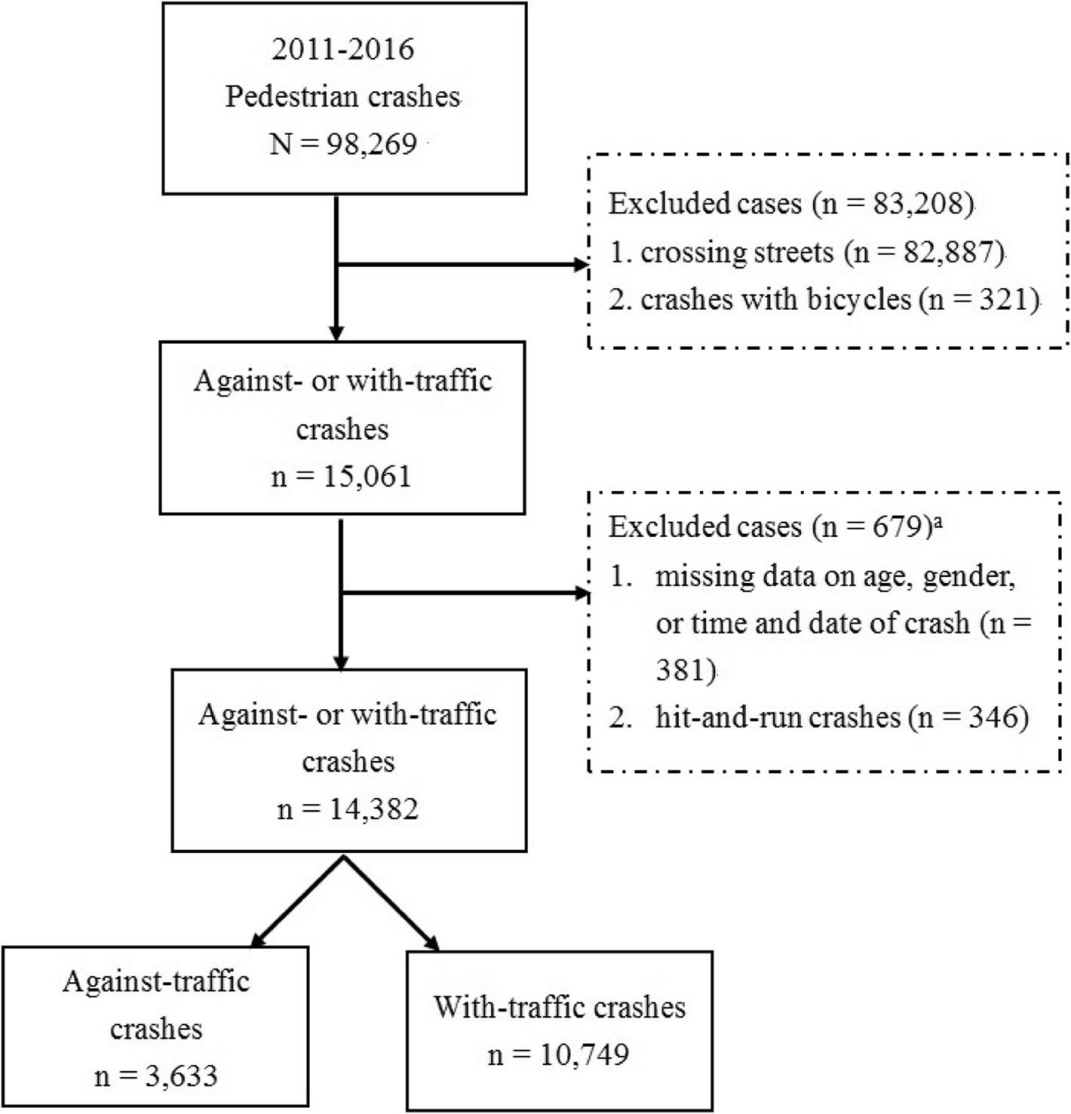
Flow chart. ^a^ Missing data and data on hit-and-run crashes were not mutually inclusive

Figure 1 presents a flow chart of the sample selection from the Taiwan National Traffic Crash Dataset for the period 2011–2016. We extracted data of 98,269 pedestrian casualties from traffic crashes during this period. We excluded crashes in which pedestrian were crossing the streets rather than walking along the streets (*n* = 83,208). Furthermore, we focused on pedestrian crashes in which the crash partner was a private car, taxi, heavy-goods vehicle, bus or coach, or a motorcycle. As a result, crashes in which pedestrians were struck with bicycles were excluded (*n* = 321). A total of 15,061 pedestrians were involved in against- or with-traffic crashes. After removing crashes with missing data on pedestrian age, sex, and time or date of crash (*n* = 679), 14,382 pedestrian causalities remained. Of the 14,382 pedestrian casualties, 3633 were walking against traffic and 10,749 were walking with traffic, respectively.

### Definitions of variables

We collected the following demographic data for casualties: sex, age (four groups: < 18, 18–40, 41–64, and ≥ 65 years), alcohol use (yes: breathalyser test results ≥0.15 mL/L or blood-alcohol consumption [BAC] level > 0.03%; no: breathalyser test results < 0.15 mL/L or BAC level ≤ 0.03%), and pedestrian walking direction (against traffic; with traffic). In Taiwan, people aged < 18 years are identified as teenagers, and they are not legally permitted to ride motorcycles or drive cars. People aged ≥65 years are identified as elderly individuals. In this study, we classified the remaining individuals into two age groups: 18–40 and 41–64 years. BAC data were obtained from police who conducted breathalyser tests or followed up for blood tests at hospitals. According to Taiwanese law, drivers with either breathalyser test results of ≥0.15 mL/L or BAC levels of > 0.03% are considered to be drunk driving. Data obtained from breathalyser tests or BAC levels were available only for motorists and not for pedestrians because, by law, only motorists involved in crashes are mandated to be tested for alcohol consumption. Data on injured body regions examined included injuries to the head or neck, upper or lower extremities, chest or abdomen, and spine.

The vehicle attribute considered was the crash partner (large vehicle: including buses, coaches, or heavy-goods vehicles; car: including private cars and taxis; and motorcycle). We examined three temporal factors, namely month of crash (spring/summer: March–August; autumn/winter: September–February), day of crash (weekday: Monday–Friday; weekend: Saturday–Sunday), and time of crash (rush hours: 0700–0859 and 1700–1859; nonrush hours: 0900–1659; evening hours: 1900–2359; and midnight/early morning: 0000–0659). The following roadway factors were considered: crash location (rural: roadways with speed limits of ≥51 km/h; urban: roadways with speed limits of ≤50 km/h), weather conditions (fine weather; adverse weather: rain or fog), street lighting condition (daylight, lit streets in darkness, and unlit streets in darkness), road surface condition (dry; slippery), and sight distance (adequate: sight distance was not obstructed; limited: sight distance was obstructed by obstacles such as road curvature, building, or tree).

### Statistical analysis

The distribution of pedestrian injury severity according to a set of variables (e.g., human attributes, roadway or environmental factors, and vehicle characteristics) is first reported. We conducted chi-squared tests to examine the association between independent variables and pedestrian injury severity. We used chi-squared tests to discover variables that were significantly associated with the outcome variables (*p* < 0.2). These variables were then incorporated into the multivariate stepwise logistic regression models. To detect multi-collinearity among the variables (all categorical), we conducted a chi-squared independent test and estimated Cramer’s V [16].

Injuries to the head, which are generally devastating, were the focus of this study. Odds of head injuries were then estimated by using stepwise logistic regression models after controlling for a set of variables.

## Results

### Pedestrian fatalities

Table 1 lists the distribution of pedestrian injury severity according to a set of variables. The mean age and standard deviation (SD) for the overall sample are 52.69 years and 21.84, and 51.24 years (SD = 22.55) for male pedestrians and 53.74 years (SD = 21.26) for female pedestrians, respectively. Of the 14,382 pedestrian casualties, 199 were fatal (1.38%) and 14,183 (98.62%) had sustained injuries. Regarding pedestrians’ walking direction, the number of fatal injuries was higher among pedestrians walking with traffic (164; 1.55%) than it was among those walking against traffic (0.88%). Notably, 7.5% of the injuries to the head or neck led to fatalities, although they were not the most frequently injured body region (*n* = 1561). The majority of the pedestrian crashes involved sober motorists (94.07%), fine weather (67.68%), rural roadways (69.95%), dry road surfaces (76.07%), fair sight distance (95.29%), walking with traffic (74.74%), motorcycles (57.2%), and weekdays (74.71%).

**Table 1:** Distribution of pedestrian injury severity according to a set of independent variables

	N	Fatality	Injury	χ^2^ test
		n (%)	n (%)	*p* value
Total	14,382 (100)	199 (1.38)	14,183 (98.62)	
Pedestrian gender				0.017
Male	6025 (41.89)	100 (1.65)	5925 (98.35)	
Female	8357 (58.11)	99 (1.18)	8258 (98.82)	
Driver gender				< 0.001
Male	9958 (69.24)	166 (1.67)	9792 (98.33)	
Female	4424 (30.76)	33 (0.75)	4391 (99.25)	
Pedestrian age				< 0.001
< 18	1079 (7.50)	3 (0.28)	1076 (99.72)	
18–40	3250 (22.60)	14 (0.43)	3236 (99.57)	
41–64	5045 (35.08)	52 (1.03)	4993 (98.97)	
≥ 65	5008 (34.82)	130 (2.60)	4878 (97.40)	
Driver age				0.882
< 18	275 (1.91)	4 (1.46)	271 (98.54)	
18–40	7912 (55.01)	109 (1.38)	7803 (98.62)	
41–64	5169 (35.94)	69 (1.34)	5100 (98.66)	
≥ 65	1026 (7.13)	17 (1.66)	1009 (98.34)	
Driver alcohol use				0.002
Yes	853 (5.93)	23 (2.70)	830 (97.30)	
No	13,529 (94.07)	176 (1.30)	13,353 (98.70)	
Months				0.225
Spring/summer	6689 (46.51)	84 (1.26)	6605 (98.74)	
Autumn/winter	7693 (53.49)	115 (1.50)	7578 (98.50)	
Weather				0.943
Fine	9734 (67.68)	134 (1.38)	9600 (98.62)	
Adverse	4648 (32.32)	65 (1.40)	4583 (98.60)	
Location				0.014
Urban (speed limit ≤50 km)	4322 (30.05)	44 (1.02)	4278 (98.98)	
Rural (speed limit > 51 km)	10,060 (69.95)	155 (1.54)	9905 (98.46)	
Light condition				0.018
Daylight	7440 (51.73)	86 (1.16)	7354 (98.84)	
Dark lit	6124 (42.58)	95 (1.55)	6026 (98.45)	
Dark unlit	818 (5.69)	18 (2.20)	800 (97.80)	
Road surface condition				0.242
Dry	10,941 (76.07)	159 (1.45)	10,782 (98.55)	
Slippery	3441 (23.93)	40 (1.16)	3401 (98.84)	
Sight distance				0.611
Adequate	13,704 (95.29)	188 (1.37)	13,516 (98.63)	
Limited	678 (4.71)	11 (1.62)	667 (98.38)	
Walking direction				0.002
With traffic	10,749 (74.74)	167 (1.55)	10,582 (98.45)	
Against traffic	3633 (25.26)	32 (0.88)	3601 (99.12)	
Crash partner				< 0.001
Large vehicle	1252 (8.70)	47 (3.75)	1205 (96.25)	
Car	4904 (34.10)	58 (1.18)	4846 (98.82)	
Motorcycle	8226 (57.20)	94 (1.14)	8132 (98.86)	
Injured body region				< 0.001
Head and neck	1561 (10.85)	117 (7.50)	1444 (92.50)	
Extremities	4013 (27.90)	2 (0.05)	4011 (99.50)	
Chest or abdomen	7955 (55.31)	72 (0.91)	7883 (99.09)	
Spine	853 (5.93)	8 (0.94)	845 (99.06)	
Day of week				0.411
Weekend	3637 (25.29)	45 (1.24)	3592 (98.76)	
Weekday	10,745 (74.71)	154 (1.43)	10,591 (98.57)	
Time of crash				< 0.001
Rush hour (0700–0859, 1700–1859)	4210 (29.27)	45 (1.07)	4165 (98.93)	
Non-rush hour (0900–1659)	4251 (29.56)	43 (1.01)	4208 (98.99)	
Evening hour (1900–2359)	4572 (31.79)	56 (1.23)	4516 (98.77)	
Midnight (0000–0659)	1349 (9.38)	55 (4.08)	1294 (95.92)	

Using chi-squared tests, we determined that the following variables were significantly associated with the outcome variable: pedestrian sex and age, driver age, driver alcohol consumption, crash location, light condition, walking direction, crash partner, injured body region, day of the week, and time of crash. These factors were then incorporated into the stepwise logistic regression models.

Table 2 presents the estimation results obtained from stepwise logistic regression models. The estimated parameter for walking with traffic was significant, suggesting that pedestrians in with-traffic crashes were 2.21 times more likely (adjusted odds ratio [AOR] = 2.21; confidence interval [CI] = 1.20–4.08) to sustain fatal injuries compared with those in against-traffic crashes. Other risk factors for fatal injuries include male drivers (AOR = 1.75; CI = 1.18–2.60), elderly pedestrians (AOR = 8.72; CI = 2.73–27.79), rural roadways (AOR = 1.37; CI = 1.04–1.80), intoxicated drivers (AOR = 1.58; CI = 1.10–2.27), unlit streets in darkness (AOR = 2.66, CI = 1.43–4.97), large vehicles as crash partners (AOR = 3.13, CI = 2.10–4.67), head or neck injuries (AOR = 6.48, CI = 3.12–13.45), and crashes occurring during midnight hours (AOR = 2.78; CI = 1.82–4.25).

**Table 2:** Odds of fatal injuries sustained by pedestrians

	β	SD	OR	95% CI	*p* value
Driver gender					
Male	0.560	0.201	1.75	1.18–2.60	0.005
Female	Ref				
Pedestrian age					
≥ 65	2.165	0.592	8.72	2.73–27.79	<.001
41–64	1.285	0.600	3.62	1.12–11.73	0.032
19–40	0.418	0.644	1.52	0.43–5.37	0.516
< =18	Ref				
Walking direction					
With traffic	0.793	0.312	2.21	1.20–4.08	< 0.001
Against traffic	Ref				
Location					
Rural	0.315	0.139	1.37	1.04–1.80	0.024
Urban	Ref				
Driver alcohol use					
Alcohol use	0.457	0.186	1.58	1.10–2.27	0.014
Alcohol non-use	Ref				
Light condition					
Dark unlit	0.979	0.319	2.66	1.43–4.97	0.002
Dark lit	0.687	0.218	1.99	1.30–3.10	0.002
Daylight	Ref				
Crash partner					
Large vehicle	1.139	0.205	3.13	2.10–4.67	<.001
Car	0.523	0.180	1.69	1.19–2.40	0.004
Motorcycle	Ref				
Injured body region					
Head and neck	1.868	0.372	6.48	3.12–13.45	<.001
Extremities	−2.882	0.793	0.056	0.012–0.27	<.001
Chest and abdomen	−0.131	0.378	0.88	0.42–1.84	0.730
Spine	Ref				
Crash of time					
Midnight	1.022	0.216	2.78	1.82–4.25	<.001
Evening hour	0.040	0.228	1.04	0.67–1.63	0.860
Non rush hour	0.170	0.249	1.19	0.73–1.93	0.494
Rush hour	Ref				

### Head or neck injuries

Because pedestrians sustaining head or neck injuries appeared to have an increased likelihood of fatalities, we further examined head or neck injuries in against-traffic crashes and with-traffic crashes, respectively. As presented in Table 3, the percentage of head or neck injuries was significantly among pedestrians in with-traffic crashes than it was among pedestrians in against-traffic crashes (11.15% vs 9.99%). Using chi-squared tests, we determined that the following variables were significantly associated with the outcome variable: pedestrian sex and age, driver sex and age, driver alcohol consumption, month of crash, weather condition, crash location, light condition, road surface condition, sight distance, walking direction, crash partner, and time of crash. These factors were then incorporated into the stepwise logistic regression models.

**Table 3:** Distribution of head or neck injury according to a set of independent variables

	N	Head and neck	Other parts	χ^2^ test
		n (%)	n (%)	*p* value
Total	14,382	1561 (10.85)	12,821 (88.15)	
Pedestrian gender				< 0.001
Male	6025 (41.89)	732 (12.15)	5302 (87.85)	
Female	8357 (58.11)	838 (10.03)	7519 (89.97)	
Driver gender				< 0.001
Male	9958 (69.24)	1147 (11.52)	8811 (88.48)	
Female	4424 (30.76)	414 (9.36)	4010 (90.64)	
Pedestrian age				< 0.001
< 18	1079 (7.50)	117 (10.84)	962 (89.16)	
18–40	3250 (22.60)	228 (7.02)	3022 (92.98)	
41–64	5045 (35.08)	548 (10.86)	4497 (89.14)	
≥ 65	5008 (34.82)	668 (13.34)	4340 (86.66)	
Driver age				0.083
< 18	275 (1.91)	36 (13.10)	239 (86.90)	
18–40	7912 (55.01)	870 (11.00)	7042 (89.00)	
41–64	5169 (35.94)	566 (10.95)	4603 (89.05)	
≥ 65	1026 (7.13)	89 (8.67)	937 (91.33)	
Driver alcohol use				0.011
Yes	853 (5.93)	115 (13.48)	738 (86.52)	
No	13,529 (94.07)	1446 (10.69)	12,083 (89.31)	
Months				0.107
Spring/summer	6689 (46.51)	696 (10.41)	5993 (89.59)	
Autumn/winter	7693 (53.49)	865 (11.24)	6828 (88.76)	
Weather				< 0.001
Good	9734 (67.68)	991 (10.18)	8743 (89.82)	
Adverse	4648 (32.32)	570 (12.26)	4078 (87.74)	
Location				0.015
Urban (speed limit <=50 km)	4322 (30.05)	419 (9.69)	3903 (90.31)	
Rural (speed limit > 51 km)	10,060 (69.95)	1112 (11.05)	8948 (88.95)	
Light condition				< 0.001
Daylight	7440 (51.73)	734 (9.87)	6706 (90.13)	
Dark lit	6124 (42.58)	694 (11.33)	5430 (88.67)	
Dark unlit	818 (5.69)	133 (16.30)	685 (83.70)	
Road surface condition				0.002
Dry	10,941 (76.07)	1137 (10.40)	9804 (89.60)	
Slippery	3441 (23.93)	434 (12.30)	3017 (87.70)	
Sight distance				0.002
Adequate	13,704 (95.29)	1463 (10.65)	12,241 (89.35)	
Limited	678 (4.71)	98 (14.55)	580 (85.55)	
Walking direction				0.050
With traffic	10,749 (74.74)	1198 (11.15)	9551 (88.85)	
Against traffic	3633 (25.26)	363 (9.99)	3270 (90.01)	
Crash partner				< 0.001
Large vehicle	1252 (8.70)	246 (19.65)	1006 (80.35)	
Car	4904 (34.10)	352 (7.18)	4552 (92.82)	
Motorcycle	8226 (57.20)	963 (11.71)	7263 (88.29)	
Day of week				0.903
Weekend	3637 (25.29)	397 (10.92)	3240 (89.08)	
Weekday	10,745 (74.71)	1164 (10.83)	9581 (89.17)	
Time of crash				< 0.001
Rush hour (0700–0859,1700–1859)	4210 (29.27)	462 (10.97)	3748 (89.03)	
Non rush hour (0900–1659)	4251 (29.56)	385 (9.06)	3866 (90.94)	
Evening hour (1900–2359)	4572((31.79)	511 (11.18)	4061 (88.82)	
Midnight (00–0659)	1349 (9.38)	203 (15.05)	1146 (84.95)	

Table 4 reports the odds of head or neck injuries as determined using multivariate stepwise logistic model. As illustrated in Table 4, pedestrians in with-traffic crashes were 1.26 times (AOR = 1.26; CI = 1.03–1.54) more likely to sustain head or neck injuries compared with those in against-traffic crashes. Other risk factors for head or neck injuries included male pedestrians (AOR = 1.18; CI = 1.06–1.31), male drivers (AOR = 1.20; CI = 1.06–1.36), elderly pedestrians (AOR = 1.30; CI = 1.05–1.61), drivers aged less than 18 (AOR = 1.50; CI = 1.06–2.12), rural roadways (AOR = 1.19; CI = 1.03–1.38), intoxicated drivers (AOR = 1.21; CI = 1.02–1.43), unlit streets in darkness (AOR = 1.59; CI = 1.25–2.01), large vehicles as crash partners (AOR = 2.03; CI = 1.75–2.41), limited sight distance (AOR = 1.24; CI = 1.02–1.51), adverse weather (AOR = 1.16; CI = 1.01–1.33), and crashes occurring during midnight or early morning hours (AOR = 1.47; CI = 1.20–1.81).

**Table 4:** Odds of head or neck injuries sustained by pedestrians

	β	SD	OR	95% CI	*P* value
Pedestrian gender					
Male	0.165	0.055	1.18	1.06–1.31	0.003
Female	Ref				
Driver sex					
Male	0.181	0.063	1.20	1.06–1.36	0.004
Female	Ref				
Pedestrian age					
≥ 65	0.265	0.108	1.30	1.05–1.61	0.015
41–64	−0.012	0.109	0.99	0.80–1.22	0.911
19–40	−0.509	0.122	0.60	0.47–0.76	< 0.001
< 18y	Ref				
Driver age					
≥ 65	Ref				
41–64	0.255	0.122	1.30	1.02–1.64	0.037
19–40	0.258	0.119	1.30	1.03–1.64	0.030
< 18y	0.405	0.176	1.50	1.06–2.12	0.027
Walking direction					
Back to traffic	0.231	0.104	1.26	1.03–1.54	0.026
Facing traffic	Ref				
Location					
Rural	0.174	0.078	1.19	1.03–1.38	0.021
Urban	Ref				
Drivers alcohol use					
Alcohol use	0.191	0.087	1.21	1.02–1.43	0.028
Alcohol non-use	Ref				
Light condition					
Dark unlit	0.461	0.122	1.59	1.25–2.01	< 0.001
Dark lit	0.119	0.084	1.13	0.96–1.33	0.115
Daylight	Ref				
Crash partner					
Large vehicle	0.710	0.087	2.03	1.72–2.41	< 0.001
Car	−0.400	0.070	0.67	0.59–0.77	< 0.001
Motorcycle	Ref				
Sight distance					
Limited	0.215	0.101	1.24	1.02–1.51	0.034
Adequate	Ref				
Weather					
Adverse	0.148	0.071	1.16	1.01–1.33	0.037
Fine	Ref				
Time of crash					
Midnight hour	0.387	0.104	1.47	1.20–1.81	< 0.001
Evening hour	0.133	0.106	1.14	0.93–1.41	0.210
Rush hour	0.123	0.083	1.13	0.96–1.33	0.139
Nonrush hour	Ref				

## Discussion

Studies have demonstrated that contraflow cycling schemes in the United Kingdom [2] and Belgium [3] decreased crash risks. Allowing contraflow cycling on one-way streets may provide cyclists and motorists with opportunities to be more alerted to each other. Considering such successful contraflow cycling schemes in Europe, the current research contributes to pedestrian safety research by concluding that in terms of crash consequence, walking against oncoming traffic is safer than walking with traffic. Coupled with the only published work conducted by Luoma and Peltola [9], who revealed a beneficial effect of walking against traffic, we recommend that all countries should consider enacting law that mandates pedestrians to walk against oncoming traffic on roadways without sidewalks.

Mian and Caird [17] conducted a laboratory study to investigate the effectiveness of retro reflectors on recognition judgments towards pedestrians standing, walking, or running. They concluded that retro-reflectively outfitted pedestrians who oriented their front to oncoming traffic were more recognisable than side-oriented or back-oriented pedestrians. In the context of our study, we recommend that, regardless of retro-reflector being used, pedestrians should orient their front to oncoming traffic.

Evidence from the existing literature [18] has shown that in approximately 60% of fatal pedestrian crashes where pedestrians were walking along roadways in Florida in year 2010, sidewalk was not available. Furthermore, Yu [19], examining the effect of built environment on severe injuries among pedestrians in Austin in USA, reported that higher sidewalk densities were associated with less severe injuries among pedestrians. Although our data do not contain information on the presence of sidewalk, it seems clear that providing pedestrians with sidewalks is a crucial intervention point to reduce crash occurrence or severity. Our results confirm prior findings that, in the event that sidewalk is not available, walking against traffic has positive implications for being less severely injuries once a crash has occurred.

Our result that with-traffic crashes were more likely to be fatal than against-traffic crashes can be explained by the higher prevalence of head injuries among pedestrians involved in with-traffic crashes. We further observed that when pedestrians were walking with traffic in unlit streets in darkness, when sight distance was obstructed, and when the weather was adverse, head injuries were more prevalent. This finding demonstrates that the negative effect of walking with traffic on head injuries is more pronounced on reduced-visibility streets. Previous studies [20–22] have established the association between unlit streets and pedestrian fatalities. Furthermore, reduced sight distance has been found to increase pedestrian crashes [23, 24]. Our results here also indicate the benefit of walking against traffic in terms of head injuries and fatalities, especially under reduced-visibility conditions. The implications of our findings here are outlines as follows. First, pedestrians should be educated to enhance their own conspicuity by using wearable reflectors under reduced-visibility conditions such as at nights, which have been reported in literature to increase detection rate and distance [17]. Second, street lighting and sight distance should be improved on roadways without sidewalks. Third, when sidewalks are absent, pedestrians should remember that they should walk against traffic, particularly under reduced-visibility conditions.

Studies have pointed out that vehicle collision velocity has the most significant effect on pedestrian head injuries, especially under a back impact [10, 11]. Congruent with these studies, we observed that head injuries tended to be more prevalent in with-traffic crashes where pedestrians were struck from behind, particularly in rural settings where collision velocities tended to be higher. Our finding thus implies that, to reduce risks of head injuries and fatalities, pedestrians should walk against traffic on roadways without sidewalks, particularly in rural settings.

The risks of head injury have been found to be substantially greater among those struck by heavy vehicles compared with those struck by cars [22, 25, 26]. The relatively high bumpers and relatively blunt frontal geometry of heavy vehicles increase the likelihood of head injuries. We contribute to the pedestrian safety literature by concluding that heavy vehicles play a crucial role in increasing both pedestrian fatalities and head injuries in with-traffic crashes. Potential countermeasures suggested in the literature include designing optimised frontal geometry configurations and energy-absorbing materials [27], which are likely to benefit pedestrians during street crossing in general and when walking along streets in particular.

Alcohol use has been consistently reported to influence driving behaviours by affecting neural processes such as cognitive abilities and reaction times, which are crucial in executing successful emergency braking and evasive manoeuvres [28, 29]. We concluded that alcohol increases the likelihood of fatal injuries and head injuries, particularly among pedestrians in with-traffic crashes. Accordingly, impaired cognitive abilities due to alcohol increase not only crash risks (as reported by Ogden and Moskowitz [30]) but also injury severity as found in our study. The fact that the majority of alcohol-related crashes occur at night necessitates tightening laws against drunk driving, especially at night, when pedestrians are less conspicuous than they are at daytime.

Our study is not without limitations. Similar to other studies that have relied on police-reported crash data, one major limitation of our study is that some crucial variables were unavailable. For example, data on intoxicated pedestrians were unavailable; alcohol use by pedestrians may play a role in injury risks, but such data were unavailable in our study. Research [31] has suggested that alcohol increased pedestrian crashes and the resulting injury severity; Fontaine and Gourlet [8] further indicated that intoxicated pedestrians were overinvolved in crashes while walking along streets. Future work may attempt to obtain additional alcohol use data from other sources. Other crucial factors that were unavailable from our data include the exact locations of pedestrian crashes. Data on the exact locations of crashes would have enabled us to obtain additional roadway characteristics such as the presence of sidewalks. A better understanding of pedestrian’s walking direction and the presence of sidewalks is a fruitful area for future research.

A noteworthy limitation of our study is that underreporting often is a problem with severity analysis. Crashes that result in injuries were more likely to be underreported than fatal crashes [32]. Recent research [33, 34] using police-reported data have mentioned these shortcomings. In the current research, however, given that the pedestrian was likely to get injured and may be likely to report the crash, the underreporting bias can be less of a concern. Our results, which were obtained by analysing police-reported crash data, should be interpreted with caution. Finally, although the injury data used in our study are generally reliable because crash investigators trace these data up to 30 days from hospitals, future work may attempt to obtain other injury data such as hospitalisation by linking our data to clinic data.

## Conclusions

We concluded that injuries to pedestrians appeared to be more severe when they were in with-traffic crashes than they were in against-traffic crashes. In addition, with-traffic crashes resulted in more head injuries than did against-traffic crashes. The negative effect of walking with traffic appeared to be more pronounced under reduce-visibility conditions such as unlit streets in darkness, adverse weather conditions, and obstructed sight distance. To reduce both crash risks and injury severity, we recommend that pedestrians should walk against traffic at roadways without sidewalks, particularly under reduced-visibility conditions.

## Data Availability

The police-reported crash data, which are open to the researchers in Taiwan, are available from the Health and Welfare Data Science Center (http://dep.mohw.gov.tw/DOS/np-2497-113.html). Only citizens of Taiwan who fulfill the requirements of conducting research projects are eligible to apply for the police-reported crash dataset. The use of police-reported crash dataset is limited to research purposes only. Applicants must follow the Computer-Processed Personal Data Protection Law.

## Abbreviations

AOR: Adjusted odds ratio
BAC: Blood-alcohol consumption
CI: Confidence interval
SD: Standard deviation
